# Supplementary material for: Clinical Features, Survival and Prognostic Factors of Glycogen-Rich Clear Cell Carcinoma (GRCC) of the Breast in the U.S. Population
Source: J Clin Med. 2019 Feb 14;8(2):246. doi: 10.3390/jcm8020246 (PMC6406344; doi:10.3390/jcm8020246)
Supplement: Supplementary file 1 [file jcm-08-00246-s001.pdf]

Supplemental Table S1. Demographical and clinical characteristics of glycogen-rich (GR) subgroup.

		GR		Non-GRCC		*p*-Value
		Count	%	Count	%	
Age	0–60 years	22	48.9%	592,600	47.3%	0.84
	>60 years	23	51.1%	659,094	52.7%	
Sex	Female	45	100.0%	1,242,754	99.3%	0.57
	Male	0	0.0%	8,940	0.7%	
Race	American Indian/ Alaska Native	0	0.0%	6,100	0.5%	0.91
	Asian or Pacific Islander	2	4.4%	85,417	6.8%	
	Black	5	11.1%	123,092	9.8%	
	Unknown	0	0.0%	5,968	0.5%	
	White	38	84.4%	1,031,117	82.4%	
Grade	Well differentiated; Grade I	3	6.7%	216,527	17.3%	**0.04**
	Moderately differentiated; Grade II	13	28.9%	446,867	35.7%	
	Poorly differentiated; Grade III	22*	48.9%	365,945	29.2%	
	Anaplastic; Grade IV	1	2.2%	16,941	1.4%	
	Unknown	6	13.3%	205,414	16.4%	
ER Status[a]	Negative	16*	36.4%	203,215	17.9%	**0.02**
	Positive	24	54.5%	807683*	71.1%	
	Borderline	0	0.0%	2,837	0.2%	
	Unknown	4	9.1%	121,615	10.7%	
PR Status[a]	Negative	23*	52.3%	311,034	27.4%	**.003**
	Positive	17	38.6%	683633*	60.2%	
	Borderline	0	0.0%	5,794	0.5%	
	Unknown	4	9.1%	134,889	11.9%	
HER2 Status[c]	Negative	12	85.7%	291,251	78.5%	0.75
	Positive	1	7.1%	52,022	14.0%	
	Borderline	0	0.0%	8,250	2.2%	
	Unknown	1	7.1%	19,360	5.2%	
IHC based intrinsic Subtype[c]	HER2-/HR+	6	42.9%	251952*	67.9%	**0.002**
	HER2+/HR-	1	7.1%	15,597	4.2%	
	HER2+/HR+	0	0.0%	36,270	9.8%	
	Triple Negative	6*	42.9%	38,757	10.4%	
	Unknown	1	7.1%	28,307	7.6%	
AJCC 6th Stage	I	14	31.1%	525,692	42.0%	0.06
	II	21*	46.7%	365,574	29.2%	
	III	5	11.1%	139,308	11.1%	
	IV	3	6.7%	54,278	4.3%	
	Unknown	2	4.4%	166,842	13.3%	
T	T0	0	0.0%	1,110	0.1%	**0.02**
	T1	17	37.8%	647,886	51.8%	
	T2	21*	46.7%	295,234	23.6%	
	T3	0	0.0%	49,240	3.9%	
	T4	2	4.4%	33,932	2.7%	
	Tis	0	0.0%	1,791	0.1%	
	Unknown	5	11.1%	222,501	17.8%	
N	N0	26	57.8%	718,263	57.4%	0.31
	N1	12	26.7%	234,469	18.7%	
	N2	3	6.7%	68,569	5.5%	
	N3	2	4.4%	48,866	3.9%	
	Unknown	2	4.4%	181,527	14.5%	
M	M0	40	88.9%	1,083,274	86.5%	0.43
	M1	3	6.7%	54,278	4.3%	
	Unknown	2	4.4%	114,142	9.1%	
Bone Metastasis[c]	No Metastasis	14	100.0%	351,808	94.9%	0.69
	Metastasis	0	0.0%	13,265	3.6%	
	Unknown	0	0.0%	5,810	1.6%	
Brain Metastasis [c]	No Metastasis	13	92.9%	363,226	97.9%	**<0.001**
	Metastasis	1*	7.1%	1,501	0.4%	

	Unknown	0	0.0%	6,156	1.7%	
Liver Metastasis[c]	No Metastasis	13	92.9%	359,808	97.0%	0.16
	Metastasis	1	7.1%	5,079	1.4%	
	Unknown	0	0.0%	5,996	1.6%	
Lung Metastasis[c]	No Metastasis	13	92.9%	358,528	96.7%	0.25
	Metastasis	1	7.1%	6,198	1.7%	
	Unknown	0	0.0%	6,157	1.7%	
Surgery[b]	No Surgery	1	2.4%	70,378	7.3%	0.42
	Surgery	40	97.6%	889,333	92.0%	
	Unknown	0	0.0%	6,824	0.7%	
Extent of Surgery[b]	No Surgery	1	2.4%	75,884	7.9%	0.55
	Sub-Mastectomy[#]	21	51.2%	485,659	50.2%	
	Mastectomy	19	46.3%	401,409	41.5%	
	Surgery, Unknown	0	0.0%	1,358	0.1%	
	Unknown	0	0.0%	2,225	0.2%	
Radiation[b]	No Radiation	20	48.8%	426,240	44.1%	0.75
	Radiation	19	46.3%	513,484	53.1%	
	Unknown	2	4.9%	26,811	2.8%	

Bolded are statistically significant p-values when comparing between clear cell/glycogen-rich to other carcinomas of the breast. [*] Statistically significant differences between column proportions. [#] Defined as any surgery less aggressive than total mastectomy. [a,b,c] Variable available for cases diagnosed after 1990, 1998 and 2010 respectively. GRCC—glycogen-rich clear cell; ER—estrogen receptor; PR—progesterone receptor; IHC—immunohistochemistry; HER2—human epidermal growth factor receptor 2; HER2+—human epidermal growth factor receptor 2 positive; HER2−—human epidermal growth factor receptor 2 negative; HR+—hormone receptor positive; HR-—hormone receptor negative; AJCC—American Joint Committee on Cancer; T, N, M refers to tumor size, nodal status, and metastasis classifications of malignant tumor staging system, respectively.

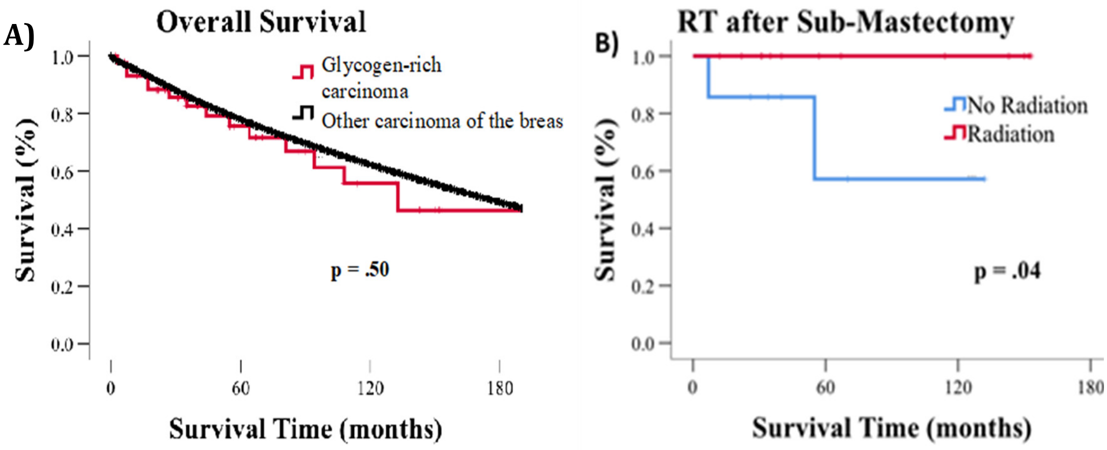

Supplemental Figure S1. Kaplan-Meier survival curves for (**A**) overall survival and (**B**) with or without radiation therapy (RT) after sub-mastectomy in the glycogen rich (GR) subgroup.

Supplemental Table S2. Survival duration in the glycogen rich (GR) subgroup.

		Months	95% CI		*p*-Value
			Lower	Upper	
All GR cases		127.5	-	-	NA
Age	60 years and under	133	96.6	219.4	0.20
	> 60 year	73	18.6	127.4	
Sex	Female	133	-	-	NA
	Male	NA	NA	NA	
Race	Asian or Pacific Islander	64	-	-	1.0
	Black	133	-	-	
	White	-	-	-	
Grade	Well to Moderately Differentiated	162	105.1	218.9	0.19
	Poorly Differentiated to Anaplastic	111	-	-	
ER Status	Negative	-	-	-	0.96
	Positive	-	-	-	
PR Status	Negative	-	-	-	0.54
	Positive	-	-	-	
AJCC Stage	I–II	-	-	-	0.12
	III–IV	94	-	-	
T	T1–2	-	-	-	0.06
	T3–4	27	-	-	
N	No nodal involvement (N0)	-	-	-	**0.02**
	Nodal Involvement (N1–3)	81	17.2	144.7	
M	M0	-	-	-	0.28
	M1	-	-	-	
Surgery	No Surgery	7	-	-	0.06
	Surgery	133	-	-	
Surgical Procedure	No Surgery	7	-	-	0.08
	Sub-Mastectomy[#]	94	65.2	122.8	
	Mastectomy	133	-	-	
Radiation	No Radiation	81	27.3	134.7	0.10
	Radiation	-	-	-	

HER2 Status was only available for cases diagnosed after 2010 and was therefore excluded from the analysis. Bolded are statistically significant p-values. "-" Median survival time was not reached or could not be calculated. [#] Defined as any surgery less aggressive than total mastectomy. CI—confidence interval; ER—estrogen receptor; PR—progesterone receptor; AJCC—American Joint Committee on Cancer; NA—not applicable; T, N, M refers to tumor size, nodal status and metastasis classifications of malignant tumor staging system, respectively.

Supplemental Table S3. Demographical and clinical characteristics of clear cell (CC) subgroup.

		CC		Non-GRCC		*p*-Value
		Count	%	Count	%	
Age	0–60 years	56	50.9%	592,544	47.3%	0.45
	>60 years	54	49.1%	659,040	52.7%	
Sex	Female	107	97.3%	1242647*	99.3%	**0.012**
	Male	3*	2.7%	8,937	0.7%	
Race	American Indian/ Alaska Native	1	0.9%	6,099	0.5%	0.77
	Asian or Pacific Islander	10	9.1%	85,407	6.8%	
	Black	11	10.0%	123,081	9.8%	
	Unknown	1	0.9%	5,967	0.5%	
	White	87	79.1%	1,031,030	82.4%	
Grade	Well differentiated; Grade I	2	1.8%	216,525	17.3%	**<0.001**
	Moderately differentiated; Grade II	40*	36.4%	446,827	35.7%	
	Poorly differentiated; Grade III	42*	38.2%	365,903	29.2%	
	Anaplastic; Grade IV	4	3.6%	16,937	1.4%	

	Unknown	22	20.0%	205,392	16.4%	
ER Status[a]	Negative	42*	42.0%	203,173	17.9%	**<0.001**
	Positive	43	43.0%	807640*	71.1%	
	Borderline	0	0.0%	2,837	0.2%	
	Unknown	15	15.0%	121,600	10.7%	
PR Status[a]	Negative	61*	61.0%	310,973	27.4%	**<0.001**
	Positive	23	23.0%	683610*	60.2%	
	Borderline	0	0.0%	5,794	0.5%	
	Unknown	16	16.0%	134,873	11.9%	
HER2 Status[c]	Negative	13	86.7%	291,238	78.5%	0.78
	Positive	1	6.7%	52,021	14.0%	
	Borderline	0	0.0%	8,250	2.2%	
	Unknown	1	6.7%	19,359	5.2%	
IHC based intrinsic Subtypes[c]	HER2-/HR+	6	40.0%	251946*	67.9%	**<0.001**
	HER2+/HR-	0	0.0%	15,597	4.2%	
	HER2+/HR+	1	6.7%	36,269	9.8%	
	Triple Negative	7*	46.7%	38,750	10.4%	
	Unknown	1	6.7%	28,306	7.6%	
AJCC 6th Stage	I	40	36.4%	525,652	42.0%	0.33
	II	38	34.5%	365,536	29.2%	
	III	12	10.9%	139,296	11.1%	
	IV	8	7.3%	54,270	4.3%	
	Unknown	12	10.9%	166,830	13.3%	
T	T0	0	0.0%	1,110	0.1%	0.16
	T1	46	41.8%	647840*	51.8%	
	T2	36*	32.7%	295,198	23.6%	
	T3	7	6.4%	49,233	3.9%	
	T4	1	0.9%	33,931	2.7%	
	Tis	0	0.0%	1,791	0.1%	
	Unknown	20	18.2%	222,481	17.8%	
N	N0	65	59.1%	718,198	57.4%	0.99
	N1	20	18.2%	234,449	18.7%	
	N2	6	5.5%	68,563	5.5%	
	N3	4	3.6%	48,862	3.9%	
	Unknown	15	13.6%	181,512	14.5%	
M	M0	92	83.6%	1,083,182	86.5%	0.32
	M1	8	7.3%	54,270	4.3%	
	Unknown	10	9.1%	114,132	9.1%	
Bone Metastasis[c]	No Metastasis	13	86.7%	351,795	94.9%	0.22
	Metastasis	1	6.7%	13,264	3.6%	
	Unknown	1	6.7%	5,809	1.6%	
Brain Metastasis[c]	No Metastasis	14	93.3%	363,212	97.9%	0.31
	Metastasis	0	0.0%	1,501	0.4%	
	Unknown	1	6.7%	6,155	1.7%	
Liver Metastasis[c]	No Metastasis	14	93.3%	359,794	97.0%	0.27
	Metastasis	0	0.0%	5,079	1.4%	
	Unknown	1	6.7%	5,995	1.6%	
Lung Metastasis[c]	No Metastasis	14	93.3%	358,514	96.7%	0.28
	Metastasis	0	0.0%	6,198	1.7%	
	Unknown	1	6.7%	6,156	1.7%	
Surgery[b]	No Surgery	6	7.0%	70,372	7.3%	0.20
	Surgery	78	90.7%	889,255	92.0%	
	Unknown	2	2.3%	6,822	0.7%	
Extent of Surgery[b]	No Surgery	7	8.1%	75,877	7.9%	0.25
	Sub-Mastectomy[#]	49	57.0%	485,610	50.2%	
	Mastectomy	29	33.7%	401,380	41.5%	
	Surgery, Unknown	0	0.0%	1,358	0.1%	
	Unknown	1	1.2%	2,224	0.2%	
Radiation[b]	No Radiation	44	51.2%	426,196	44.1%	0.35

Radiation	39	45.3%	513,445	53.1%
Unknown	3	3.5%	26,808	2.8%

Bolded are statistically significant *p*-values when comparing clear cell carcinoma to other carcinomas of the breast. * Statistically significant differences between column proportions. # Defined as any surgery less aggressive than total mastectomy. [a,b,c] Variable available for cases diagnosed after 1990, 1998 and 2010 respectively. GRCC—glycogen-rich clear cell; ER—estrogen receptor; PR—progesterone receptor; IHC—immunohistochemistry; HER2—human epidermal growth factor receptor 2; HER2+—human epidermal growth factor receptor 2 positive; HER2−—human epidermal growth factor receptor 2 negative; HR+—hormone receptor positive; HR−—hormone receptor negative; AJCC—American Joint Committee on Cancer; T, N, M refers to tumor size, nodal status and metastasis classifications of malignant tumor staging system, respectively.

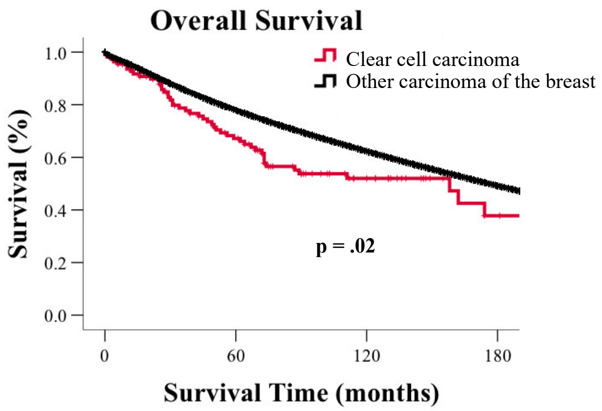

Supplemental Figure S2. Kaplan-Meier survival curves for clear cell (CC) subgroup.

Supplemental Table S4. Survival duration in the clear cell (CC) subgroup.

		Months	95% CI		*p*-Value
			Lower	Upper	
All Cases		158	80.6	235.4	NA
Age	60 years and under	73	63.6	82.4	**0.006**
	>60 year	158	80.6	235.4	
Sex	Female	158	80.9	235.1	0.62
	Male	73	18.6	127.4	
Race	American Indian/Alaska Native	27	-	-	0.17
	Asian or Pacific Islander	-	-	-	
	Black	-	-	-	
	White	111	27.7	194.3	
Grade	Well to Moderately Differentiated	162	69.2	254.8	0.16
	Poorly Differentiated to Anaplastic	111	-	-	
ER Status	Negative	158	73.7	242.3	0.82
	Positive	174	49.5	298.5	
PR Status	Negative	158	18.5	297.5	0.22
	Positive	-	-	-	
AJCC Stage	I–II	174	150.2	197.8	**<0.001**
	III–IV	37	11.0	63.0	
T	T1–2	162	139.9	184.1	**0.04**
	T3–4	27	21.9	32.1	
N	No nodal involvement (N0)	174	150.0	198.0	**0.02**
	Nodal Involvement (N1–3)	69	-	-	
M	M0	162	78.7	245.3	**<0.001**
	M1	30	0.0	80.1	
Surgery	No Surgery	25	0.0	59.0	**<0.001**

	Surgery	162	97.3	226.7	
	No Surgery	25	-	-	
Extent of Surgery	Sub-Mastectomy	174	54.5	293.5	**0.05**
	Mastectomy	-	-	-	
Radiation	No Radiation	73	2.8	143.2	**0.05**
	Radiation	162	-	-	

HER2 Status was only available for cases diagnosed after 2010 and was therefore excluded from the analysis. Bolded are statistically significant p-values. "-" Median survival time was not reached or could not be calculated. [#] Defined as any surgery less aggressive than total mastectomy. CI—confidence interval; ER—estrogen receptor; PR—progesterone receptor; AJCC—American Joint Committee on Cancer; NA—not applicable; T, N, M refers to tumor size, nodal status and metastasis classifications of malignant tumor staging system, respectively.

Supplemental Table S5. Multivariable analysis of overall survival for clear cell (CC) carcinomas.

Variable	Reference	*p*-Value	HR	95% CI	
				Lower	Upper
Age		**<0.001**	1.05	1.02	1.07
AJCC Stage (III IV)	I–II	**<0.001**	5.47	2.23	13.41
Surgery	No Surgery	0.58	0.67	0.16	2.78
Radiation	No Radiation	0.11	0.55	0.27	1.14

Bolded are statistically significant p-values. CI—confidence interval; HR—hazards ratio; AJCC—American Joint Committee on Cancer.

Supplemental Table S6. Comparison of demographical and clinical characteristics between glycogen-rich (GR) and clear cell (CC) carcinomas.

		GR		CC		*p*-Value
		Count	%	Count	%	
Age	0–60 years	22	48.9%	56	50.9%	0.82
	>60 years	23	51.1%	54	49.1%	
Sex	Female	45	100.0%	107	97.3%	0.26
	Male	0	0.0%	3	2.7%	
Race	American Indian/ Alaska Native	0	0.0%	1	0.9%	0.76
	Asian or Pacific Islander	2	4.4%	10	9.1%	
	Black	5	11.1%	11	10.0%	
	Unknown	0	0.0%	1	0.9%	
	White	38	84.4%	87	79.1%	
Grade	Well differentiated; Grade I	3	6.7%	2	1.8%	0.32
	Moderately differentiated; Grade II	13	28.9%	40	36.4%	
	Poorly differentiated; Grade III	22	48.9%	42	38.2%	
	Anaplastic; Grade IV	1	2.2%	4	3.6%	
	Unknown	6	13.3%	22	20.0%	
ER Status[a]	Negative	16	36.4%	42	42.0%	0.38
	Positive	24	54.5%	43	43.0%	
	Borderline	0	0.0%	0	0.0%	
	Unknown	4	9.1%	15	15.0%	
PR Status[a]	Negative	23	52.3%	61	61.0%	0.13
	Positive	17	38.6%	23	23.0%	
	Borderline	0	0.0%	0	0.0%	
	Unknown	4	9.1%	16	16.0%	
HER2 Status[c]	Negative	12	85.7%	13	86.7%	0.99
	Positive	1	7.1%	1	6.7%	
	Borderline	0	0.0%	0	0.0%	
	Unknown	1	7.1%	1	6.7%	
IHC based intrinsic Subtypes[c]	HER2-/HR+	6	42.9%	6	40.0%	0.73
	HER2+/HR-	1	7.1%	0	0.0%	
	HER2+/HR+	0	0.0%	1	6.7%	

	Triple Negative	6	42.9%	7	46.7%	
	Unknown	1	7.1%	1	6.7%	
AJCC 6th Stage	I	14	31.1%	40	36.4%	0.56
	II	21	46.7%	38	34.5%	
	III	5	11.1%	12	10.9%	
	IV	3	6.7%	8	7.3%	
	Unknown	2	4.4%	12	10.9%	
T	T0	0	0.0%	0	0.0%	0.10
	T1	17	37.8%	46	41.8%	
	T2	21	46.7%	36	32.7%	
	T3	0	0.0%	7	6.4%	
	T4	2	4.4%	1	0.9%	
	Tis	0	0.0%	0	0.0%	
	Unknown	5	11.1%	20	18.2%	
N	N0	26	57.8%	65	59.1%	0.45
	N1	12	26.7%	20	18.2%	
	N2	3	6.7%	6	5.5%	
	N3	2	4.4%	4	3.6%	
	Unknown	2	4.4%	15	13.6%	
M	M0	40	88.9%	92	83.6%	0.60
	M1	3	6.7%	8	7.3%	
	Unknown	2	4.4%	10	9.1%	
Bone Metastasis[c]	No Metastasis	14	100.0%	13	86.7%	0.37
	Metastasis	0	0.0%	1	6.7%	
	Unknown	0	0.0%	1	6.7%	
Brain Metastasis[c]	No Metastasis	13	92.9%	14	93.3%	0.37
	Metastasis	1	7.1%	0	0.0%	
	Unknown	0	0.0%	1	6.7%	
Liver Metastasis[c]	No Metastasis	13	92.9%	14	93.3%	0.37
	Metastasis	1	7.1%	0	0.0%	
	Unknown	0	0.0%	1	6.7%	
Lung Metastasis[c]	No Metastasis	13	92.9%	14	93.3%	0.37
	Metastasis	1	7.1%	0	0.0%	
	Unknown	0	0.0%	1	6.7%	
Surgery[b]	No Surgery	1	2.4%	6	7.0%	0.34
	Surgery	40	97.6%	78	90.7%	
	Unknown	0	0.0%	2	2.3%	
Extent of Surgery[b]	No Surgery	1	2.4%	7	8.1%	0.36
	Sub-Mastectomy[#]	21	51.2%	49	57.0%	
	Mastectomy	19	46.3%	29	33.7%	
	Surgery, Unknown	0	0.0%	0	0.0%	
	Unknown	0	0.0%	1	1.2%	
Radiation[b]	No Radiation	20	48.8%	44	51.2%	0.92
	Radiation	19	46.3%	39	45.3%	
	Unknown	2	4.9%	3	3.5%	

Bolded are statistically significant p-values when comparing between glycogen-rich carcinoma with clear cell carcinoma. [*] Statistically significant differences between column proportions. [#] Defined as any surgery less aggressive than total mastectomy. [a,b,c] Variable available for cases diagnosed after 1990, 1998 and 2010 respectively. ER—estrogen receptor; PR—progesterone receptor; IHC—immunohistochemistry; HER2—human epidermal growth factor receptor 2; HER2+—human epidermal growth factor receptor 2 positive; HER2−—human epidermal growth factor receptor 2 negative; HR+—hormone receptor positive; HR−—hormone receptor negative; AJCC—American Joint Committee on Cancer; T, N, M refers to tumor size, nodal status and metastasis classifications of malignant tumor staging system, respectively.

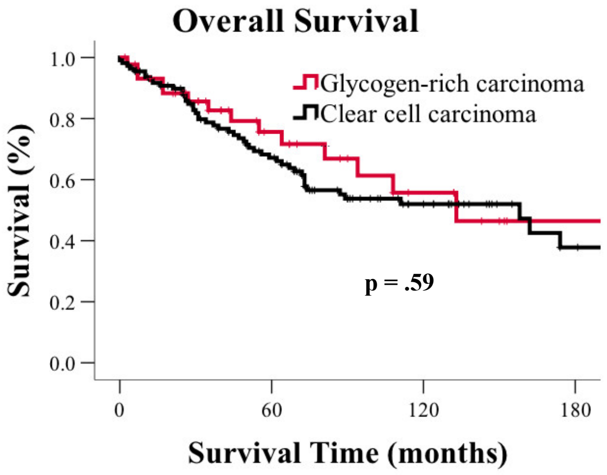

Supplemental Figure S3. Kaplan-Meier survival curves comparing glycogen-rich (GR) and clear cell (CC) carcinomas.
